# Prevalence and determinants of physical activity behavior in a peri-urban population in Nepal: A secondary analysis of the Dhulikhel Heart Study

**DOI:** 10.1525/agh.2025.2476634

**Published:** 2025-10-18

**Authors:** Neha Balapal, Margaret Chi, Dinesh Timalsena, Lindy Reynolds, Niroj Bhandari, Archana Shrestha

**Affiliations:** 1 City University of New York School of Medicine, New York, NY, USA; 2 University of Washington, Seattle, WA, USA; 3 Kathmandu University School of Medical Sciences/Dhulikhel Hospital, Dhulikhel, Nepal; 4 Pratyanashil Community Development Society (PRAYAS Nepal), Dhading, Nepal; 5 University of Alabama at Birmingham, Birmingham, AL, USA

**Keywords:** Physical activity, Cardiovascular disease, Leisure time physical activity, Sedentary behavior

## Abstract

Physical inactivity is a well-known modifiable risk factor for the prevention and treatment of leading noncommunicable diseases. Secondary data analysis was performed using cross-sectional self-reported 2014 and 2023 data from the Dhulikhel Heart Study which assesses cardiovascular disease risk factors and outcomes in a peri-urban population of Nepal. Physical activity questions in the Dhulikhel Heart Study were assessed using the Global Physical Activity Questionnaire. Our analysis found that 82% of the study sample met the World Health Organization requirements for adequate physical activity and the average daily sedentary behavior was 34 min/day. However, there were high self-reported occupational physical activity and low leisure time physical activity rates among all demographic groups. However, there were high self-reported occupational physical activity and low leisure time physical activity rates among all demographic groups. The multivariable analysis found that age (OR = 1.26, 95% CI = 1.13–1.41), unemployment (OR = 2.22, 95% CI = 1.55–3.16), anxiety (OR = 3.33, 95% CI = 1.24–4.26), depression (OR = 1.71, 95% CI = 1.09–2.63), and comorbidity (OR = 2.62, 95% CI = 1.55–3.29) were all significantly associated with lower physical activity. Lack of formal education (OR = 0.51, 95% CI = 0.30–0.87), alcohol consumption (OR = 0.56, 95% CI = 0.33–0.97), central obesity (OR = 0.58, 95% CI = 0.36–0.92), and moderate and high perceived stress (OR = 0.19, 95% CI = 0.12–0.29) were significantly associated with medium and high physical activity. Therefore, the total self-reported physical activity in our study population is high, but still lower than findings from the national representative 2019 WHO STEPwise approach to surveillance Survey. Low leisure time physical activity is also a concern in our population, a finding consistent with other studies in South Asia. Future studies should examine factors related to leisure time physical activity and the effect of physical activity interventions on overall health in peri-urban populations in Nepal.

## Background

The majority of annual noncommunicable disease (NCD) deaths globally arise from cardiovascular disease (CVD) (17.9 million), making CVD prevention and treatment a leading priority for health agencies and governments around the world [1]. Physical inactivity, with an estimated global prevalence of 31%, is a well-known modifiable risk factor for preventing and treating leading NCDs such as CVD [2]. It is currently the fourth leading cause of death in NCDs through its contributions to stroke, coronary artery disease, and type 2 diabetes mellitus [3–5]. Furthermore, adequate physical activity (PA) can majorly benefit overall physical, psychological, social, and cognitive health [6, 7]. For the purpose of implementing health measures, especially in low- and middle-income countries, PA is often measured in domains of day-to-day life: occupational, transportational, and leisure time physical activity (LTPA) [2, 4]. The occupational domain consists of PA that exists at work, especially manual labor, or domestic labor required for the home. The transportational domain refers to PA related to transport including commuting to the workplace (walking, cycling) or to other locations for daily activities of living. The leisure time domain encompasses PA done in free time such as recreational activities, sports, or social activities [2, 4]. Transportation-related and LTPA, in particular, have been shown to improve health, decrease the risk of all-cause mortality, reduce sickness-related occupational absences, and prevent NCDs like CVD, diabetes, and cancer [8–12]. Although these benefits are well-established, it is estimated that 1 in 4 adults and 3 in 4 adolescents do not currently meet the World Health Organization’s (WHO) PA recommendation of at least 60 min/day of moderate intensity or equivalent PA for adolescents and at least 150 min/week of moderate intensity or equivalent PA for adults [13]. As the global pandemic of physical inactivity continues to rise, it becomes imperative to advance research and interventions that can mitigate its health and economic consequences [14].

The majority of adults in South Asia do not engage in LTPA, and Nepal is no exception to this [15]. The trend of insufficient PA among adults has increased from 2.4% to 7.4% from 2013 to 2019 and only 7.3% of total PA comes from recreational activities [16]. Current PA data in Nepal from WHO STEPwise approach to surveillance (STEPS) surveys have focused predominantly on urban areas [15]. Furthermore, residing in urban settings have been found to be associated with lower PA levels in Nepal [16]. Urbanization has led to an economic shift from manual labor to sedentary occupations resulting in a trend of decreasing occupational PA [17, 18], although travel and work-related activity is still the leading domain of PA for people in Nepal [19]. Cultural influences such as family responsibilities, schedule, social embarrassment, and lack of familial support can deter people from engaging in PA [19], and a lack of public spaces like parks and playgrounds can be a major barrier to access [20]. Most PA in Nepal is performed due to routine activities: women primarily engage in household activities, and men engage in active commuting and agricultural work. However, people with diagnosed chronic diseases are more likely to engage in PA for the benefit of their health, and education about the importance of PA can be a facilitator for increasing PA engagement [17, 19]. As transportation-related PA decreases as ownership of private vehicles and sedentary occupations become more widespread [17], there is a need to explore the PA behaviors of people in Nepal to design appropriate individual and community-level interventions. Nepal does not currently have national, comprehensive guidelines on PA, therefore, clinicians and researchers rely on the WHO recommendations for PA. Existing studies show that overall PA is still low in some parts of Nepal [21]. Nepal is a geographically diverse country, with sociodemographic characteristics and health outcomes varying between communities. To create a multisector approach for decreasing sedentary behavior in Nepal, the patterns and prevalence of various domains of PA need to be characterized in the context of specific Nepalese populations.

In this detailed secondary analysis, we utilized data from the Dhulikhel Heart Study (DHS) to estimate sedentary behavior and PA patterns and determine the sociodemographic and CVD risk factors associated with low levels of PA in the suburban population of Dhulikhel [22]. Findings from prior studies indicate that there is a high burden of CVD risk factors in the Dhulikhel population [23]. In this study, we evaluated the association of sociodemographic, behavioral, and clinical factors with PA levels to better understand the public health burden of low PA in this setting. In addition, we sought to quantify the sedentary behavior levels in this population to inform future community-based activities.

## Methods

### Study design

The DHS is a population-based, prospective cohort study that started in 2013 and focuses on CVD risk factors in a suburban town in central Nepal [23]. In 2023, the second wave of the DHS was implemented from August 2022 to August 2023. All eligible participants residing in the sample households were invited to participate. The eligibility criteria included being a permanent resident of Dhulikhel and residing there physically for at least 6 months, 18 years of age or older, nonpregnant, and ability to communicate in a local language. A total of 1,609 eligible participants have been enrolled during the 2 waves of recruitment in 2014 and 2023. Trained research assistants conducted in-person interviews at participant’s households and directly entered the data into a tablet. Data were stored in a RedCap database [24]. Ethical approval for the DHS was obtained from the institutional review board of Kathmandu University School of Medical Sciences (Approval number 63/13). A total of 1,507 participants with available data on PA were included in the current analyses. Of the 1,507 participants included in this analysis, 81% (*n* = 1,216) are from wave 1 and 19% (*n* = 291) are from wave 2.

### Variables

The DHS questionnaire consists of questions from various standardized tools. Sociodemographic variables including age (completed years), sex (male, female), ethnicity (Brahmin/Chhetri, Newar, Others), marital status (married or cohabitating, not currently married or separated), income (wealth quintile), and education (no formal education, less than high school, high school or more) are adopted from the WHO STEPS survey [16]. “Others” in the ethnicity category consists of Janajati and Dalit ethnicities. Those ever married or cohabitating were categorized in the “married or cohabitating” group, whereas those single, widowed, or separated were categorized as “not currently married or separated.” Education was collected as completed schooling years. Ten to 12 completed years of schooling constitutes a high school education. Wealth quintiles were determined using data collected in the Household Questionnaire (Demographic Health Survey wealth index) based on household assets such as vehicle ownership, dwelling characteristics, toilet facilities, drinking water sources, and other material assets related to wealth status [25]. Principal Component Analysis method was leveraged using these household asset variables to calculate the wealth quintiles. Quintile 1 represents the least wealthy quintile of the DHS study population, whereas quintile 5 represents the most wealthy. Lifestyle variables assessed included tobacco use (Global Adult Tobacco Survey Questionnaire) [26], alcohol consumption (self-developed tool), diet (Diet Quality Questionnaire; DQQ) [27], stress (Perceived Stress Scale; PSS) [28], depression (Patient Health Questionnaire 9; PHQ–9) [29], and anxiety (Generalized Anxiety Disorder 7; GAD–7) [30]. Tobacco use was categorized as current, former, and never smokers. Alcohol use was categorized as current, former, and never drinkers. The Global Dietary Recommendations (GDR) score was calculated from the DQQ and is a measure of alignment with WHO global recommendations for healthy diets. GDR scores were categorized as “adequate” (score of 10 or higher) to indicate meeting at least 50% of the WHO dietary recommendations and “inadequate” (score less than 10), indicating not meeting at least 50% of the WHO dietary recommendations. Responses to the PHQ–9 were categorized as “depression” (score of 9 or higher) and “no depression” (score less than 9) [31]. Responses to the GAD–7 questionnaire were categorized as “anxiety” (score of 7 or higher) and “no anxiety” (score less than 7) [32]. Perceived stress scores from the PSS were categorized as low (0–13), moderate (14–26), and high (27–40) stress.

Physical activity questions were ascertained using Global Physical Activity Questionnaire (GPAQ). The GPAQ assesses self-reported PA during work, transport, and leisure time over a typical week for a participant [33]. The questionnaire also assesses sedentary behavior, defined as activities that include energy expenditure at a level of 1.0 to 1.5 metabolic equivalent time (MET) [34]. The GPAQ has been used to assess PA patterns in several other populations in Nepal [35], and it has been indicated as a valid and reliable tool for assessing PA [36]. Physical activity was reported through MET in min/week. The total PA was reported by calculating the sum of PA across all domains (occupational, transportation-related, and leisure time). Total PA score is categorized as low (<600 MET-min/week), moderate (600–3,000 MET-min/week), and high (>3,000 MET-min/week) for descriptive analysis. These categories were selected to evaluate participants based on meeting the WHO recommendation for >600 MET-min/week of PA reflected by moderate or high PA levels.

Measured health characteristics from the DHS included pulse (beats per minute), waist circumference (cm), hip circumference (cm), height (cm), and weight (kg). Body mass index (BMI) was calculated as the ratio between body mass (kg) and body height squared (m^2^) and was categorized as underweight (<18.5 kg/m^2^), normal weight (18.5–24.9 kg/m^2^), overweight (25–29.9 kg/m^2^), and obese (≥30 kg/m^2^) [37]. Blood pressure was measured using the average of 3 measurements of systolic and diastolic blood pressure (mm Hg) taken using a Microlife automatic blood pressure measuring device. Hypertension was defined as having a mean systolic blood pressure ≥140 mm Hg or a diastolic blood pressure of ≥90 mm Hg or those who were receiving antihypertensive medication. Medical history questions were self-reported diseases diagnosed by a physician. Health-related determinant characteristics included in the analysis were BMI, waist-to-hip ratio, hypertension, diabetes, anxiety, and multimorbidity. Central obesity was defined as waist-to-hip ratio of ≥0.9 for males and ≥0.85 for females. Multimorbidity was defined as reporting 2 or more of the following: hypertension, diabetes, myocardial infarction, stroke, obesity, depression, anxiety, chronic obstructive pulmonary disease/asthma, kidney disease, peptic ulcer disease, atrial fibrillation, congestive heart failure, rheumatic heart diseases, deep vein thrombosis, pulmonary embolism, liver diseases, and cancer or malignant tumor.

### Data analysis

Descriptive statistics are reported as frequencies and percentages for categorical variables, and mean and standard deviation (SD) were reported for continuous variables. The descriptive characteristics between the 2 waves of enrollment were also compared. Physical activity was calculated for each of the 3 PA domains (work, leisure-time, and transportation-related) using the metabolic equivalent (MET)-minutes per week. This was done by multiplying the minutes spent for various forms of activity by the relevant metabolic equivalent intensity factor. This was further multiplied by the number of days per week the participant partook in these activities. The domain scores were summed to calculate the total PA score and then categorized as low, moderate, and high. We conducted logistic regression with low PA as outcome and sociodemographic and health-related variables as independent variables to determine the associations between sociodemographic and health-related variables and PA. Statistical significance in Table 3 was calculated for each variable (e.g., age, sex, marital status, etc.) within a specific PA type (e.g., occupational, transportation-related, etc.). A two-sample *t*-test for binary variables and a one-way ANOVA for variables with 3 or more categories. All statistical analyses were carried out in R (v4.3.2, R Core Team 2023).

## Results

A comparison of sociodemographic and health behavior characteristics between wave 1 and wave 2 are shown in Table 1. There were no statistically significant differences between wave 1 and wave 2 in the characteristics of sex, age, hypertension, and education. Among wave 2 participants there was an increase in the not currently married marital status, an increase in Brahmin ethnicity, a decrease in Newar ethnicity, an increase in the “student/nonpaid/other” occupation, an increase in normal weight BMI, and a decrease in overweight BMI (*P* < .001). The sociodemographic and health behavior characteristics of the study participants are presented in Table 2. The overall prevalence of low, moderate, and high PA in this population was 18.6%, 25.6%, and 55.8%, respectively. The sample included 60% women. The average age of the sample population was 46 years (SD = 17). The participants engaging in low PA were older (51). Most participants were married or cohabitating (76.9%), and most were employed (60.2%). However, 42.9% of participants were less than high school educated. The most prevalent ethnic groups were Brahmin/Chhetri (45%) and Newar (31%). Among those with low PA, most people were in the employed group (47%), married or cohabitating group (90%), of Brahmin/Chhetri ethnicity (40.0%), from wealth quintile 3 (24.9%), had no formal education or less than high school education (37.1%), never users of tobacco (81.5%), and never drinkers (85.6%). Those with low PA were also mostly with no diabetes (90.0%), no depression (81.8%), no multimorbidity (73.9%), and moderate stress (40.7%). Among those with moderate PA, most people were employed (54.4%), married or cohabitating (72.8%), of Brahmin/Chhetri ethnicity (42.2%), from wealth quintile 5 (25.3%), had less than high school education (40.5%), never users of alcohol (82%), and never drinkers (80.4%). They also were mostly with no diabetes (87.8%), no depression (89.1%), no multimorbidity (81.6%), and high stress (53.5%). Among those with high PA, most people were employed (67.3%), married or cohabitating (81.2%), of Brahmin/Chhetri ethnicity (48.2%), from wealth quintile 1 (23.1%), had less than high school education (45.8%), never users of tobacco (78.3%), and never drinkers (80.7%). They also were mostly with no diabetes (93.7%), no depression (87.3%), no multimorbidity (86.1%), and high stress (49%).

Table 3 presents the mean and SD of MET-min/week by sociodemographic characteristics of the participants. The median total self-reported PA was 3,360 (IQR = 7,020) MET-min/week. Self-reported MET-min/week were highest in the occupational PA domain across all demographic categories. Leisure time physical activity was lower across all categories, particularly among women, those over age 70, homemaker/retired occupations, and those with no formal education compared to occupational and transportational PA. The median overall LTPA was 0 MET-min/week, and total LTPA among the study population accounted for less than 2% of total PA (not shown). Average sedentary behavior per week was 1,666 min (SD = 896), which averages 238 min/day.

Sociodemographic determinants of low PA are shown in Table 4. The odds of low PA compared to moderate/high PA was higher by 26% (OR = 1.26, 95% CI = 1.13–1.41) for every 10 years increase in age. Unemployed participants had higher odds of low PA compared to the employed (OR = 2.22, 95% CI = 1.55–3.16). Those with no formal education had lower odds of low PA compared to those with high school education or more (OR = 0.51, 95% CI = 0.30–0.87), even after adjusting for sex, marital status, occupation, ethnicity, education, and wealth quartile.

Lifestyle determinants of low PA compared to moderate/high PA are shown in Table 5 with an adjusted model that includes tobacco use, alcohol use, GDR score, perceived stress score, multimorbidity, and all sociodemographic variables. Current alcohol drinkers have half the odds of low PA than never drinkers (OR = 0.56, 95% CI = 0.33–0.97). Those with PSS scores indicating high (OR = 0.19, 95% CI = 0.12–0.29) or moderate (OR = 0.26, 95%CI = 0.17–0.40) stress had lower odds of low PA than those with low stress. Using tobacco and diet quality did not show significant association with low PA.

Table 6 shows the health characteristic determinants of low PA compared to moderate/high PA with an adjusted model that includes all sociodemographic variables, BMI, waist-to-hip ratio, multimorbidity, and lifestyle variables (tobacco use, alcohol use, GDR score, perceived stress). The odds of low PA compared to moderate/high PA was lower among those with central obesity (OR = 0.58, 95% CI = 0.36–0.92) compared to those with a normal waist-to-hip ratio. The odds of low PA were higher among those with self-reported hypertension (OR = 4.10, 95% CI = 1.47–10.79), depression (OR = 1.71, 95% CI = 1.09–2.63), anxiety (OR = 2.33, 95% CI = 1.24–4.26), and multimorbidity (OR = 2.62, 95% CI = 1.55–3.29) compared to their respective comparison groups.

## Discussion

This cross-sectional secondary analysis provides baseline information about PA patterns and determinants among Nepalese adults in Dhulikhel, Nepal. Overall, this population was shown to have a high prevalence of adequate PA (82%) represented by moderate or high PA (>600 MET-min/week). In addition, more than half of the population met the requirements for high PA (56%). Increased likelihood of engaging in low PA was associated with increasing age, unemployed status, positive hypertension status, positive depression status, positive anxiety status, and presence of multimorbidity. The prevalence of those meeting adequate WHO PA recommendations in our study is high compared to other countries [38]. In India, the prevalence of people who were physically active was about 50% [39–41], whereas the prevalence among Peruvian adults was 62% [42]. However, our findings are consistent with other community-based studies in Nepal, which have found prevalences ranging from 78% to 82% [42, 43]. This finding is much lower than that of a nationally representative study covering urban and rural populations in Nepal, which found a prevalence of 97% [35]. The prevalence of adequate PA in our study population is also lower than the 92.6% found in the 2019 WHO Nepal STEPS survey [16]. Previous discussions regarding the overall high prevalence of adequate PA in Nepal have suggested that Nepal’s agrarian culture, reliance on walking for commuting due to lack of transportation infrastructure, and over-reporting of PA levels have contributed to this finding [35].

The average amount of sedentary behavior (238 min/day) was slightly higher in our study population than in a similar study conducted in Western Nepal (179.8 min/day) [17], and the WHO 2019 STEPS survey reported 201.2 min/day [16]. It is possible that this is due to Dhulikhel municipality’s geographic proximity to the very urban capital Kathmandu. As many residents may travel to neighboring urban settings for occupation, they may engage in higher sedentary behavior related to commuting and office-related occupation settings. This is corroborated by the work of Paudel et al. [18] who found that in Nepal, urban populations engage in less occupational, transport, and total PA overall as compared to rural populations.

Our study found low amounts of LTPA compared to occupational and transportation PA, which aligns with previous studies on PA habits of Nepalese adults and other studies in South Asia [15, 44]. These PA patterns in Nepal and the broader South Asian region could be attributed to agrarian societies where it is common for people to engage in PA while they tend to their farms, and lack of transportation infrastructure contributing to the high amounts of transportation-related PA as people often must walk or ride a bicycle to get from place to place.

Our multivariable model found higher odds of engaging in low PA among unemployed people. This may be explained by the likelihood that unemployed population members may be more likely to do house-related work and/or engage in sedentary behaviors. According to the Government of Nepal National Population and Housing Census, reasons given for census members not engaged in economic activity in Dhulikhel include household work, household chores, age, disability, pension, and social work. It is possible that our unemployed study population may not be physically able to partake in PA, or engage in activities that do not correspond with occupational or transportation-related PA [45].

We also found several medical history factors associated with low PA in our study, including hypertension, depression, anxiety, and multimorbidity. Previous studies have shown a bidirectional relationship between medical problems such as hypertension or multimorbidity and low PA, which may also explain our findings [46–48]. It is well-established that people with low PA are more likely to develop medical complications later in life [49, 50]. In addition, many people with existing medical problems or chronic disease are not able to engage in PA due to chronic fatigue or loss of functional capacity [49]. Mental illness (particularly depression and anxiety) also has a known bidirectional mutually reinforcing relationship with low PA [51, 52]. People with depression or anxiety are known to have lower participation in PA due to a myriad of factors, including high sedentary behavior, low motivation and confidence to exercise, low self-efficacy, and lack of social support for exercise [53].

Factors that contributed toward moderate to high PA in our study include no formal education, moderate or high perceived stress, increased waist-to-hip ratio/central obesity, and current alcohol drinkers. Our finding of the inverse relationship between education level and PA is consistent with prior studies in Nepal [35, 54]. One potential explanation is that people with higher education levels tend to have more sedentary office jobs, whereas people with no formal education are more likely to have jobs that are physically demanding. A prior study in a peri-urban community in a different part of Nepal also found lower levels of physical inactivity in more educated participants [54]. Both males and females with higher education levels had lower PA even after adjusting for age, marital status, urban or rural status, smoking status, and BMI [35].

Additionally, our study noted that people who reported moderate- to high-stress levels are more likely to engage in PA than those who reported low stress levels. This could be explained by the fact that people may engage in PA to combat their stress levels, which has been noted in prior studies on the effects of stress on PA [55]. A systematic review on the topic found that 18.2% of prospective studies noted that PA was positively impacted by stress and posited that this is because some individuals utilize exercise to cope with stress which aligns with the findings from our study [55]. Alternatively, this association may be linked to socioeconomic status as people from a low socioeconomic background can have higher levels of stress related to uncertainty and increased life stressors. A multinational study evaluating the relationship between sedentary behavior and perceived stress found an association between increased sedentary behavior and increased perceived stress, however the directionality was not confirmed. They hypothesized that wealth, education, and unemployment may play a role in this relationship [56]. Interestingly, our study noted that people with an increased waist-to-hip ratio/central obesity had higher odds of reporting moderate to high PA compared to their normal counterparts. We hypothesize that this is because people with central obesity may engage in more PA in efforts to lose abdominal fat for either aesthetic or health promoting reasons. It is possible that these people have been told by a health-care professional they should exercise to lose weight or faced social and/or cultural pressures to lose weight through PA. Alternatively, participants may be overreporting their PA to indicate they are trying to lose weight (social desirability bias). Finally, people in our study who were current drinkers were more likely to report higher levels of PA which aligns with the findings from a previous study conducted in Nepal [54]. Although our analysis adjusted for age, this could be due to the fact that over 50% of the people who reported currently drinking in our study were younger than 50 years (residual confounding). As age increases, people are more likely to engage in low amounts of PA. As such, we think the observed association between current drinkers and moderate to high PA could partially be attributed to residual confounding by age.

### Strengths and limitations

Our analysis adds valuable information to the literature on characteristics associated with the amount and types of PA in a diverse, peri-urban community in central Nepal that has not been previously studied, which can aid in developing effective interventions to address one of the main risk factors for the development of CVD in Nepal and other low- and middle-income settings. In addition, we obtained high-quality data due to the use of previously validated tools for most of the data collection, and our data analysis was thorough in accounting for important confounders. Several limitations to our study must be noted. Our study was limited to one municipality in the Kavrepalanchowk district. Given that Nepal is an ethnically diverse country, ethnic group distributions vary based on municipality. Our study has a high percentage of Newari participants (31%) compared to the national reported average (5%), as some parts of Dhulikhel are predominantly Newari communities [57]. Therefore, the study results may not be representative of other parts of Nepal. Another limitation was that sample collection from wave 1 to wave 2 was delayed due to COVID-19, resulting in a change from sampling household data to individual people data in wave 2. Furthermore, the adoption of Nepal’s new federal constitution in 2015 led to significant administrative restructuring across the country, including changes in municipal boundaries and governance structures. As a result, the sampling frame of our study was affected. In 2013, Dhulikhel Municipality consisted of 9 wards; however, following federalization, 3 additional wards were incorporated, bringing the total to 12. This administrative shift has implications for comparability across survey waves, as the population and geographic scope of the study area expanded over time [58]. To explore potential differences in sample population, we have included a comparison between wave 1 and wave 2 sociodemographic and health-related characteristics in Table 1. In addition to political changes, a major earthquake in 2015 directly impacted our study site in Nepal, causing delays in enrollment. These delays were further compounded by the COVID-19 pandemic. Although we pooled the data from the 2 waves for analysis, we made adjustments for measured covariates. Despite this, residual confounding due to unmeasured or imperfectly measured variables may still bias the results. Additionally, differences in underlying contextual or environmental factors between the 2 time periods beyond what was captured in the sociodemographic data may influence outcomes in ways not accounted for in the analysis.

Physical activity, particularly LTPA, is a multifaceted behavioral phenomenon that has numerous influences including individual behavior, community, cultural structure, environmental factors, as well as local government and policy, all of which cannot be accounted for through quantitative research methods alone. This cross-sectional analysis cannot adequately account for these influences so some residual confounding may remain in the adjusted associations. However, the variables included in our adjusted models have been noted in prior studies to be significantly associated with PA levels in Nepalese populations so the effect of unmeasured variables in this study is most likely minimal [54, 44]. In addition, PA was self-reported by participants in this study which introduces the potential for recall and social desirability bias. The use of a previously validated data collection tool, the GPAQ, limits the potential for these biases to have affected our study results since the tool assesses PA over the past 1 week (short recall period) of the interview time [59]. Prior studies have shown the GPAQ, a tool developed by the WHO, to be a valid and reliable tool for measuring PA and sedentary behavior in certain South Asian populations [60–62]. The regression models for Tables 4, 5, and 6 were adjusted by a different set of confounders such that we only adjusted for the relevant covariates to obtain a minimally biased estimate. The authors recognize that forward and backward selection models can be considered in this context, however when running these models, the value of the odds ratio did not change to the other side of the null indicating it does not significantly change our regression results. Therefore, minimal sufficient adjustment was used. Finally, this analysis was based on cross-sectional data, so no causal inferences can be made from these results.

### Study implications

While our study showed that 82% of this peri-urban community in central Nepal had adequate PA, our results also showed this population had low amounts of LTPA compared to occupational or transportation-related PA. The results from this study will direct future qualitative studies that examine barriers and facilitators for LTPA. Specifically, conducting in-depth interviews or focus group discussions with participants from different ethnic groups, education levels, gender, and marital status would yield valuable insight that could inform the development of tailored interventions to increase LTPA in this population. The results from these studies could be used to design community-based activities that specifically encourage the groups of people in our study who engage in low amounts of LTPA to participate. Focusing on these populations will aid in increasing LTPA in our community which could help address one of the most important risk factors for CVD and the rising burden of CVD in our community. Because the DHS is an ongoing study that tracks participants for up to 20 years, future studies among DHS participants will allow us to better understand the relationship between low amounts of LTPA and the prevention of or development of poor CVD-related health outcomes. Understanding the reasons for engaging in low amounts of LTPA and the relationship between low LTPA and various CVD outcomes would aid in identifying strengths in the current risk factor counseling practices and preventive health programs in this community as well as areas for improvement. The goal of this line of research is to facilitate engagement in LTPA, inform health-care resource allocation to improve CVD-related health outcomes in a community where the burden of CVD is rising and resources are limited, as well as to inform policies and guidelines on PA at the district, provincial, and national levels in Nepal which do not currently exist. We recommend that the results of this study be used to inform community education materials and the organization of district wide PA intervention efforts. Given the low prevalence of leisure-time PA found in this population, concentrated community efforts to engage the population in recreational PA could improve physical and mental well-being.

## Conclusions

This study conducted a secondary data analysis of cross-sectional DHS data to evaluate socioeconomic and health history related determinants of PA levels and domains among adults in a peri-urban community in Nepal. There was a high overall prevalence of PA in our study population but low levels of LTPA. Determinants of PA level in this population included age, education level, employment status, alcohol use status, perceived stress, waist-to-hip .ratio, multimorbidity, and history of hypertension, depression, and anxiety (*P* < 0.05). As urbanization continues to expand globally, occupational PA may no longer be an adequate source of physical activity for the Nepalese population. Leisure time PA specifically is important for cardiorespiratory fitness, regardless of occupational PA [63], and plays a key role in conferring positive mental health [64]. Encouraging PA behavior in Nepal is a complex issue that requires both community-level programs and structural and policy-based approaches. Therefore, consolidated efforts to provide community-level education on the health-related benefits of PA and encourage LTPA are paramount.

## Figures and Tables

**Table 1.** Sociodemographic and health-related characteristics of participants with low, moderate, and high physical activity based on enrollment group (*N* = 1,507)^a^

	Enrollment Group, *n* (%)		
Characteristics	Wave 1 *n* = 291 (19.3)	Wave 2 *n* = 1,216 (80.7)	*P* value
Sex, *n* (%)			0.788
Male	123 (42.3)	488 (40.1)	
Female	168 (57.7)	728 (59.9)	
Age, mean (SD), years	51 (15)	45 (17)	0.548
Marital status, *n* (%)			**<0.001**
Not currently married or separated	45 (15.5)	303 (24.9)	
Married or cohabitating	246 (84.5)	913 (75.1)	
Occupation, *n* (%)			**<0.001**
Employed	211 (72.5)	696 (57.3)	
Unemployed	60 (20.6)	237 (19.5)	
Student/nonpaid/others	5 (1.7)	129 (10.6)	
Homemaker/retired	15 (5.2)	153 (12.6)	
Ethnicity, *n* (%)			**<0.001**
Brahmin/Chhetri	67 (23.0)	613 (50.4)	
Newar	174 (59.8)	289 (23.8)	
Others^b^	50 (17.2)	314 (25.8)	
Education, *n* (%)			0.297
No formal education	106 (36.4)	396 (33.0)	
Less than high school	113 (38.8)	526 (43.8)	
High school or more	72 (24.7)	278 (23.2)	
Body mass index, *n* (%)			**<0.001**
Underweight	8 (2.9)	69 (5.9)	
Normal weight	94 (33.9)	570 (48.3)	
Overweight	116 (41.9)	391 (33.2)	
Obese	59 (21.3)	149 (12.6)	
Hypertension, *n* (%)			
No	285 (98.3)	1,195 (98.5)	0.974
Yes	5 (1.7)	18 (1.5)	
Multimorbidities, *n* (%)			**0.037**
No multimorbidity	228 (78.4)	1,018 (83.7)	
Multimorbidity	63 (21.6)	198 (16.3)	

a Bold values indicate *P* <0.05. Statistical significance was determined using Chi-squared test for categorical variables and a one-way ANOVA for continuous variables. Percentages reported relative to column attribute.

b Other category for ethnicity includes Dalits and Janajatis.

**Table 2.** Sociodemographic and health-related characteristics of participants with low, moderate, and high physical activity (*N* = 1,507)^a^

	Physical Activity, *n* (%)				
Characteristics	Low *n* = 280 (18.6)	Moderate *n* = 386 (25.6)	High *n* = 841 (55.8)	Total	*P* value
Sex, *n* (%)					0.265
Male	109 (38.9)	170 (44.0)	332 (39.5)	611 (40.5)	
Female	171 (61.1)	216 (56.0)	509 (60.5)	896 (59.5)	
Age, mean (SD), years	51 (21)	47 (18)	45 (15)	46 (17)	**<0.001**
Marital status, *n* (%)					**<0.001**
Not currently married or separated	85 (30.4)	105 (27.2)	158 (18.8)	348 (23.1)	
Married or cohabitating	195 (69.6)	281 (72.8)	683 (81.2)	1,159 (76.9)	
Occupation, *n* (%)					**<0.001**
Employed	131 (47.0)	210 (54.4)	566 (67.3)	907 (60.2)	
Unemployed	88 (31.5)	92 (23.8)	117 (13.9)	297 (19.7)	
Student/nonpaid/others	22 (7.9)	50 (13.0)	62 (7.4)	134 (8.9)	
Homemaker/retired	38 (13.6)	34 (8.8)	96 (11.4)	168 (11.2)	
Ethnicity, *n* (%)					**<0.001**
Brahmin/Chhetri	112 (40.0)	163 (42.2)	405 (48.2)	680 (45.1)	
Newar	102 (36.4)	149 (38.6)	212 (25.2)	463 (30.7)	
Others^b^	66 (23.6)	74 (19.2)	224 (26.6)	366 (24.2)	
Wealth quintile					**<0.001**
Quintile 1	46 (17.1)	60 (16.1)	185 (23.1)	291 (20.2)	
Quintile 2	47 (17.5)	62 (16.7)	183 (22.8)	292 (20.2)	
Quintile 3	67 (24.9)	74 (19.9)	150 (19)	289 (20.2)	
Quintile 4	47 (17.5)	82 (22.0)	156 (20)	285 (19.8)	
Quintile 5	62 (23.0)	94 (25.3)	124 (16)	286 (19.8)	
Education, *n* (%)					**0.015**
No formal education	101 (37,1)	121 (31.6)	280 (33.5)	502 (33.7)	
Less than high school	101 (37.1)	155 (40.5)	383 (45.8)	639 (42.9)	
High school or more	70 (25.7)	107 (27.9)	173 (20.7)	350 (23.5)	
Tobacco user, *n* (%)					**0.002**
Current user	37 (14.0)	50 (14.0)	160 (20.2)	247 (17.5)	
Former user	12 (4.5)	14 (3.9)	12 (1.5)	38 (2.7)	
Never user	2,163 (81.5)	292 (82.0)	622 (78.3)	1,130 79.9)	
Alcohol, *n* (%)					**0.024**
Current drinker	28.0 (10.5)	64 (16.7)	143 (17.2)	235 (15.7)	
Former drinker	12 (4.3)	11 (2.9)	18 (2.2)	41 (2.7)	
Never drinker	238 (85.6)	308 (80.4)	672 (80.7)	1,218 (81.5)	
Global Dietary Recommendations Score (0–18)	11.7 (1.6)	11.9 (1.7)	11.8 (1.6)	11.8 (1.6)	0.383
Body mass index, *n* (%)					0.135
Underweight	15 (5.6)	16 (4.3)	46 (5.6)	77 (5.3)	
Normal weight	119 (44.9)	151 (40.7)	393 (48.0)	664 (45.6)	
Overweight	88 (33.0)	150 (40.4)	269 (32.9)	507 (34.8)	
Obese	44 (16.5)	54 (14.6)	110 (13.4)	208 (14.3)	
Mean BMI (SD)	25.4 (4.6)	25.6 (4.3)	24.9 (4.3)	25.2 (4.3)	0.051
Waist-to-hip ratio, *n* (%)					0.187
Normal	155 (56.2)	220 (57.1)	436 (52.0)	811 (54.1)	
Increased	121 (43.8)	165 (42.9)	402 (48.0)	688 (45.9)	
Hypertension, *n* (%)					
No	271 (97.1)	380 (99.0)	829 (98.7)	1,480 (98.5)	0.123
Yes	8 (2.9)	4 (1.0)	11 (1.3)	23 (1.5)	
Diabetes, *n* (%)					**0.002**
No	252 (90.0)	339 (87.8)	788 (93.7)	1,379 (91.5)	
Yes	28 (10.0)	46 (12.2)	53 (6.3)	128 (8.5)	
Depression screening, *n* (%)					**0.018**
No depression	229 (81.8)	344 (89.1)	734 (87.3)	1,307 (86.7)	
Depression	51 (18.2)	42 (10.9)	107 (12.7)	200 (13.3)	
Anxiety screening, *n* (%)					0.096
No anxiety	260 (92.9)	372 (96.4)	803 (95.5)	1,435 (95.2)	
Anxiety	20 (7.1)	14 (3.6)	38 (4.5)	72 (4.8)	
Multimorbidities, *n* (%)					**<0.001**
No multimorbidity	207 (73.9)	315 (81.6)	724 (86.1)	1,246 (82.7)	
Multimorbidity	73 (26.1)	71 (18.4)	117 (13.9)	261 (17.3)	
Perceived stress scale, *n* (%)					**<0.001**
Low stress (0–13)	62.0 (22.1)	39.0 (10.1)	68 (8.1)	169 (11.2)	
Moderate stress (14–26)	114 (40.7)	141 (36.5)	361 (42.9)	616 (40.9)	
High stress (27–40)	104 (37.1)	206 (53.4)	412 (49.0)	722 (47.9)	

a Bold values indicate *P* <0.05. Statistical significance was determined using Chi-squared test for categorical variables and a one-way ANOVA for continuous variables. Percentages reported relative to column attribute.

b Other category for ethnicity includes Dalits and Janajatis.

**Table 3.** MET-minutes of domains of physical activity and minutes of sedentary behavior (*N* = 1.507)^a^

	Median (IQR) of Physical Activity (MET-min/week)				Mean (SD) of Sedentary Behavior (min/week)
	Occupational PA	Transportation PA	Leisure-Time PA	Total	
Total	1,680 (6,480)	560 (1,680)	0 (0)	5,938 (7,335)	1,666 (896)
Sex					
Male	1,680 (6,720)	1,433 (2,289)	0 (0)	**6,101 (7,616)**	**1,728 (921)**
Female	1,680 (6,130)	1,373 (2,501)	0 (0)	**5,827 (7,139)**	**1,623 (876)**
Age					
18–29	**1,120 (5,040)**	**480 (1,680)**	0 (0)	**3,360 (5,880)**	**1,656 (856)**
30–39	**2,080 (7,500)**	**720 (1,680)**	0 (0)	**5,040 (8,400)**	**1,524 (892)**
40–49	**3,480 (7,900)**	**840 (1,680)**	0 (0)	**5,940 (8,580)**	**1,441 (838)**
50–59	**1,840 (6,720)**	**840 (2,520)**	0 (0)	**4,140 (7,200)**	**1,561 (799)**
60–69	**840 (4,800)**	**560 (1,680)**	0 (0)	**3,360 (5,040)**	**1,823 (919)**
70+	**0 (1,680)**	**80 (960)**	0 (0)	**840 (3,360)**	**1,823 (919)**
Marital status					
Not currently married or separated	**290 (3,840)**	**480 (1,680)**	0 (0)	**1,920 (4,590)**	**1,715 (833)**
Married or cohabitating	**1,960 (6,720)**	**720 (1,680)**	0 (0)	**4,080 (7,280)**	**1,659 (904)**
Occupation					
Employed	**3,240 (7,920)**	**720 (1,680)**	0 (0)	**5,040 (8,400)**	**1,531 (885)**
Unemployed	**0 (3,360)**	**420 (1,680)**	0 (0)	**1,680 (3,780)**	**2,070 (893)**
Student/nonpaid/others	**420 (3,360)**	**840 (1,680)**	0 (0)	**2,460 (4,145)**	**1,627 (742)**
Homemaker/retire	**3,140 (6,720)**	**0 (1,260)**	0 (0)	**3,360 (6,720)**	**1,704 (863)**
Ethnicity					
Brahmin/Chhetri	**2,100 (6,720)**	**560 (1,680)**	0 (0)	**3,880 (7,440)**	**1,657 (859)**
Newar	**700 (3,360)**	**720 (1,680)**	0 (0)	**2,340 (5,040)**	**1,729 (929)**
Other	**3,360 (6,720)**	**560 (1,680)**	0 (0)	**4,520 (7,155)**	**1,601 (918)**
Education					
High school or more	**960 (5,040)**	**560 (1,680)**	0 (0)	**2,910 (5,880)**	**1,681 (866)**
Less than high school	**1,920 (6,720)**	**840 (1,680)**	0 (0)	**4,320 (7,140)**	**1,598 (879)**
No formal education	**1,680 (6,180)**	**560 (1,680)**	0 (0)	**3,360 (7,530)**	**1,739 (936)**
Wealth quintile					
Quintile 1	**3,360 (8,400)**	**480 (1,680)**	0 (0)	**5,040 (8,400)**	**1,585 (801)**
Quintile 2	**3,360 (6,720)**	**720 (1,680)**	0 (0)	**5,040 (6,720)**	**1,620 (943)**
Quintile 3	**1,120 (5,760)**	**420 (1,680)**	0 (0)	**3,080 (5,880)**	**1,699 (865)**
Quintile 4	**1,120 (5,040)**	**840 (1,680)**	0 (0)	**3,360 (7,560)**	**1,673 (932)**
Quintile 5	**560 (3,710)**	**720 (1,680)**	0 (0)	**2,390 (4,920)**	**1,742 (906)**
Body mass index					
Normal weight	2,400 (6,720)	560 (1,680)	0 (0)	3,880 (7,280)	1,677 (874)
Underweight	1,680 (6,240)	0 (1,680)	0 (0)	3,360 (7,080)	1,737 (935)
Overweight	1,120 (5,400)	840 (1,680)	0 (0)	3,360 (7,110)	1,612 (902)
Obese	1,350 (5,040)	390 (1,680)	0 (0)	3,360 (5,880)	1,713 (935)
Tobacco user					
Never user	**1,680 (6,000)**	**560 (1,680)**	0 (0)	**3,360 (6,915)**	**1,632 (854)**
Former user	**280 (2,640)**	**280 (840)**	0 (0)	**1,340 (2,980)**	**2,130 (1,026)**
Current user	**3,360 (7,200)**	**560 (1,680)**	0 (0)	**5,040 (7,080)**	**1,641 (943)**
Alcohol use					
Never drinker	1,680 (6,480)	**560 (1,680)**	0 (0)	3,360 (7,440)	1,646 (877)
Former drinker	840 (5,040)	**0 (960)**	0 (0)	2,160 (6,160)	2,097 (1,224)
Current drinker	1,680 (6,120)	**840 (1,680)**	0 (0)	3,600 (6,300)	1,694 (917)
Global Dietary Recommendations Score					
Adequate	1,680 (6,440)	720 (1,680)	0 (0)	3,360 (7,080)	**1,648 (892)**
Inadequate	1,040 (6,720)	280 (1,680)	0 (0)	3,360 (6,600)	**1,854 (920)**
Diabetes					
No diabetes	**1,680 (6,720)**	560 (1,680)	0 (0)	**3,600 (7,440)**	1,662 (896)
Diabetes	**0 (1,680)**	840 (1,785)	0 (0)	**1,680 (3,510)**	1,706 (892)
Hypertension					
No	1,680 (6,480)	600 (1,680)	0 (0)	3,360 (7,020)	1,665 (894)
Yes	840 (7,680)	420 (2,100)	0 (0)	1,680 (8,070)	1,561 (1,015)
Waist-to-hip ratio					
Normal	1,440 (6,720)	**700 (1,680)**	0 (0)	3,360 (7,500)	1,675 (912)
Central obesity	1,920 (6,240)	**560 (1,680)**	0 (0)	3,540 (6,510)	1,652 (876)

a Bold values indicate *P* <0.05. Statistical significance was calculated by column and variable using two-sample *t*-test for binary variables and a one-way ANOVA for variables with 3 or more categories.

**Table 4.** Sociodemographic determinants of low physical activity (LPA) compared to moderate to high physical activity (MHPA) among DHS participants (*N* = 1,507)^a^

Characteristics	Unadjusted Odds Ratio for LPA vs. MHPA (95% CI)	Adjusted Odds Ratio for LPA vs. MHPA (95% CI)
Sex		
Male	1	1
Female	1.09 (0.83–1.42)	1.12 (0.82–1.55)
Age, mean^b^	**1.23 (1.14–1.32)**	**1.26 (1.13–1.41)**
Marital status		
Married or cohabitating	1	1
Not currently married or separated	**1.60 (1.19–2.13)**	**1.26 (0.89–1.74)**
Occupation		
Employed	1	1
Unemployed	**2.50 (1.83–3.40)**	**2.03 (1.42–2.88)**
Student/nonpaid/others	1.16 (0.70–1.87)	1.03 (0.57–1.79)
Homemaker/retired	**1.73 (1.14–2.58)**	**1.40 (0.88–2.20)**
Ethnicity		
Brahmin/Chhetri	1	1
Newar	**1.43 (1.06–1.93)**	1.30 (0.93–1.81)
Others	1.12 (0.80–1.57)	1.39 (0.96–2.01)
Wealth quintile		
Quintile 5	1	1
Quintile 1	0.68 (0.44–1.03)	0.76 (0.47–1.22)
Quintile 2	0.69 (0.45–1.05)	0.82 (0.52–1.29)
Quintile 3	1.09 (0.74–1.62)	1.22 (0.80–1.85)
Quintile 4	0.71 (0.47–1.09)	0.81 (0.52–1.27)
Education		
High school or more	1	1
No formal education	1.01 (0.72–1.42)	**0.51 (0.30–0.87)**
Less than high school	0.75 (0.54–1.06)	0.68 (0.45–1.02)

a Bold values indicate *P* <0.05. Statistical significance was determined using the Wald test. Adjusted for all sociodemographic variables (sex, age, marital status, occupation, ethnicity, education, and wealth quintile).

b Increment for age is 10 years

**Table 5.** Lifestyle determinants of low physical activity (LPA) compared to moderate to high physical activity (MHPA) among DHS participants (*N* = 1,507)^a^

Characteristics	Unadjusted Odds Ratio for LPA vs. MHPA (95% CI)	Adjusted Odds Ratio for LPA vs. MHPA (95% CI)
Tobacco user		
Never user	1	1
Current user	0.76 (0.51–1.10)	0.91 (0.57–1.42)
Former user	1.97 (0.95–3.89)	1.72 (0.74–3.84)
Alcohol		
Never drinker	1	1
Current drinker	**0.51 (0.33–0.78)**	**0.56 (0.33–0.97)**
Former drinker	1.70 (0.82–3.29)	1.47 (0.56–3.60)
Global Dietary Recommendations Score		
Adequate	1	1
Inadequate	1.08 (0.67–1.67)	0.85 (0.48–1.45)
Perceived stress scale		
Low stress (0–13)	1	1
Moderate stress (14–26)	**0.39 (0.27–0.56)**	**0.26 (0.17–0.40)**
High stress (27–40)	**0.29 (0.20–0.42)**	**0.19 (0.12–0.29)**

a Bold values indicate *P* <0.05. Statistical significance was determined using the Wald test. Adjusted for all sociodemographic variables, tobacco use, alcohol use, GDR score, perceived stress score, and multimorbidity.

**Table 6.** Health-related determinants of low physical activity (LPA) compared to moderate to high physical activity (MHPA) among DHS participants (*N* = 1,507)^a^

Characteristics	Unadjusted odds ratio for LPA vs. MHPA (95% CI)	Adjusted odds ratio for LPA vs. MHPA (95% CI)
Body mass index		
Normal weight	1	1
Underweight	1.10 (0.58–1.95)	1.14 (0.54–2.26)
Overweight	0.94 (0.69–1.27)	0.80 (0.56–1.15)
Obese	1.23 (0.83–1.81)	1.18 (0.75–1.85)
Mean BMI^b^	1.01 (0.98–1.04)	1.00 (0.96–1.04)
Waist-to-hip ratio		
Normal	1	1
Central obesity	0.91 (0.69–1.18)	**0.58 (0.36–0.92)**
Hypertension		
No hypertension	1	1
Hypertension	**2.39 (0.95–5.56)**	**4.10 (1.47–10.79)**
Diabetes		
No diabetes	1	1
Diabetes	1.26 (0.80–1.93)	0.77 (0.44–1.32)
Depression screening		
No depression	1	1
Depression	**1.63 (1.44–2.30)**	**1.71 (1.09–2.63)**
Anxiety screening		
No anxiety	1	1
Anxiety	**1.75 (1.00–2.93)**	**2.33 (1.24–4.26)**
Multimorbidities		
No multimorbidity	1	1
Multimorbidity	**1.93 (1.41–2.62)**	**2.62 (1.55–3.29)**

a Bold values indicate *P* <0.05. Statistical significance was determined using the Wald test. Adjusted for sociodemographic variables, BMI, waist-to-hip ratio, multimorbidity, and lifestyle variables (tobacco use, alcohol use, GDR score, and perceived stress).

b Increment for BMI is 1 kg/m^2^.

## Data Availability

The authors will make deidentified study data available upon request.
